# Healthy Eating and Active Lifestyle after Bowel Cancer (HEAL ABC)—feasibility randomised controlled trial

**DOI:** 10.1038/s41430-024-01491-z

**Published:** 2024-08-27

**Authors:** Jana Sremanakova, Anne Marie Sowerbutts, Chris Todd, Richard Cooke, Lyndsay Pearce, David Leiberman, John McLaughlin, Jim Hill, Helen Ashby, Aswatha Ramesh, Sorrel Burden

**Affiliations:** 1 School of Health Sciences, University of Manchester, Oxford Road, Manchester, M13 9PL UK; 2 Manchester Academic Health Science Centre, Manchester, M13 9PL UK; 3 NIHR Applied Research Collaboration Greater Manchester, Manchester, M13 9NQ UK; 4 Manchester University Hospitals NHS Foundation Trust, Manchester, M13 9WL UK; 5 Department of Psychology, School of Health, Education, Policing and Sciences, Staffordshire University, Stoke-on-Trent, ST4 2DE UK; 6 Salford Royal NHS Foundation Trust, Manchester, M6 8HD UK; 7 School of Medical Sciences, University of Manchester, Oxford Road, Manchester, M13 9PL UK

**Keywords:** Cancer prevention, Nutrition

## Abstract

**Background:**

Evidence from cohort studies indicates that a healthy lifestyle can improve cancer survival but evidence from randomised controlled trials (RCT) is lacking. Thus, this study tested the feasibility of conducting a lifestyle intervention in patients after colorectal cancer (CRC) treatment.

**Methods:**

An intervention was developed based on World Cancer Research Fund and American Institute for Cancer Research (WCRF/AICR) recommendations, the Health Action Process Approach, Motivational Interviewing and tested a feasibility, mixed-methods RCT. Participants were allocated to a three-month telephone-based intervention versus standard care control group. The follow up period was six months. Data on feasibility and secondary outcomes were collected and analysed using Stata (V15, StataCorp LLC) and NVivo 12 (QSR International Pty Ltd., Doncaster, VIC).

**Results:**

Recruitment was challenging (31 ineligible, 37 declined; recruitment rate = 48.6%.). In total, 34/35 participants completed the intervention, and 31 (89%) completed follow up; all 31 completers participated in six telephone calls during intervention and six months follow up. Study retention was 97% (34/35) and 89% (31/35) at three and six months, respectively. Data completion rates were high (>90%). Intervention was acceptable to participants, met their needs and kept them accountable towards their goals. Participants in the intervention group showed significant improvement in WCRF/AICR, Diet Quality Index-International score and a 10% reduction in ultra-processed food consumption.

**Conclusions:**

The HEAL ABC intervention was feasible for 87% of intervention participants, supporting them in healthy lifestyle changes. However, alternative recruitment strategies are needed for a fully powered RCT to determine the effectiveness of the intervention.

## Introduction

Colorectal cancer (CRC) is estimated to increase to 3.2 million new cases and 1.6 million deaths annually by 2040 [1]. Although, survival rates are increasing due to advances in cancer care [2], quality of life can be compromised by post-treatment morbidities and disease recurrence [3].

The European Prospective Investigation into Cancer and Nutrition (EPIC) study [4] and the World Cancer Research Fund and American Institute for Cancer Research (WCRF/AICR) Continuous Update Project [5, 6] both demonstrated the importance of adopting a healthy lifestyle (including maintaining a healthy body weight and following dietary and physical activity prevention recommendations) for primary cancer prevention. Several studies showed that the prevention recommendations formulated as part of WCRF/AICR Second Expert Report [6] could reduce the risk of cancer recurrence [7–9]. However, most healthcare organisations do not have funding [10], training or personnel [11] to translate this into clinical practice. In addition, studies have not yet demonstrated a benefit of long-term adherence to pragmatic cancer prevention recommendations [12]. Cochrane report [13] showed weak to moderate evidence that dietary interventions led to behaviour change at 3 to 12 months post-treatment and highlighted the need for long-term follow-up studies, particularly in other survivor groups than breast cancer.

There are few RCTs after colorectal cancer (CRC) treatment and limited evidence of the effect of dietary interventions [14]. Patients with and beyond CRC do try to make physical activity [15] and dietary changes [16]. However, these changes are often insignificant without professional support [16]. There are patient preferences for advice communication [17] and priorities [18, 19]. It was suggested that when designing behavioural change studies, it is critical to identify the “active ingredients” that drive change and thus incorporate behavioural change theory in the intervention [20, 21]. Previous studies have focused on the effectiveness of an intervention [13, 22], rather than which aspect of the intervention contributed to the changes observed [23]. As such, there is a lack of true understanding about how interventions work, what are the active ingredients, and which interventions lead to effective long-term adherence to cancer prevention recommendations. Hence, the aim of this research was to assess the feasibility of Healthy Eating and Active Lifestyle After Bowel Cancer (HEAL ABC) intervention to be performed as a fully powered RCT.

## Materials and methods

### Trial design

HEAL ABC trial is a mixed-method, feasibility, parallel-group RCT using 1:1 randomisation. This study followed MRC framework [20], and CONSORT guidelines [24] and the Template for Intervention Description and Replication (TIDieR) check list. The study was approved by the North West Greater Manchester South Research Ethics Committee (IRAS ID 273818), and registered on the National Institute of Health Clinical Trials register (NCT04227353). Trial protocol was published [25]. The COVID-19 global pandemic resulted in some deviations from the original study protocol. Assessments were conducted over the telephone, anthropometry measurements were self-reported, body composition assessments were not undertaken, and medical records of participants recruited via the public domain could not be viewed.

### Study population and recruitment

Adults (age ≥ 18 years), a minimum of 12 weeks after CRC surgery with all treatment complete following less than four of the WCRF/AICR recommendations were eligible for inclusion into the study. In the hospitals, appropriate participants were identified from clinical records. Participants were recruited from three cancer centres in Great Manchester. Also, research was advertised in the public domain across England, and thus potential participants could directly contact researcher about study participation. Written informed consent was obtained, and baseline data collection followed the randomisation process.

### Intervention

The HEAL ABC trial intervention was based on WCRF/AICR prevention recommendations [6], following the principles of Schwarzer’s (1998) Health Action Process Approach (HAPA) theory [26], and telephone calls were informed by Motivation Interviewing Technique (MIT) [27]. Details on theory implementation in the intervention [25] and HEAL ABC resource development [18] were previously published. Behaviour change techniques used are presented as Supplement 1. Participants randomised to the control group received a leaflet by post with links to healthy lifestyle recommendations available online (Supplement 2). No additional support was provided. The intervention was delivered by researcher (as part of PhD project) who is qualified nutritionist, holds fitness trainer level 2 qualification and completed training in Motivation Interviewing Technique.

### Data collection

Participants were assessed at baseline, three months post intervention, and six month follow up. The main outcomes were recruitment, retention, attrition, and data completion rates. Adherence to the intervention was assessed by looking at completion of intervention calls, the time to complete the whole study including six months follow up and number of goals achieved. The goal was considered achieved if it had been practiced from the time the goal was set until the three months assessment. Participants verbally confirmed their actions or practice during every intervention call; and these calls were audio recorded. Also, participants were asked about their confidence of making the changes they had set for themselves during the intervention, and success rate at the end of the study. Intervention feasibility was evaluated using the Shanyinde 2011 criteria [28]. The acceptability of the intervention was explored qualitatively through interviews conducted at three-months post intervention and at six months follow up.

Adherence to the WCRF/AICR recommendations was assessed by using the WCRF/AICR standardised scoring system [29]. Data on anthropometry, dietary intake (three-day food diaries and INTAKE24 software [30]), physical activity (Global Physical Activity Questionnaire—GPAQ [31]), Yamx Digi walker model CW-701 (YAMASA TOKEI KEIKI CO., LTD, Japan) and sociodemographic (questionnaire) were collected. At six months, cancer recurrence, morbidity, and survival rates were collected. Nutrients were assessed alongside food groups including—fruit, vegetables, fruit and vegetables (grams/day and portions/day), wholegrains (grams/day), red and processed meat (grams per week). Ultra-processed food (UPFs) was calculated as a percentage of energy from the total energy intake based on Nova classification [32], and added sugar calculated in teaspoons per day. In addition, Diet Quality Index International [33] was calculated.

### Interviews

Sampling—convenience sampling was used and participants were selected at baseline, as the first 12 to 15 until data saturation recommended by Guest et al. [34]. Positioning—the researcher conducting the intervention interviewed the participants as part of the PhD training. The researcher developed friendly relationships with the participants and established trust with them. This is important as it allowed to get rich data, but participants might have been overly positive about the intervention. The participants’ logs were kept with notes and reflections from telephone calls. All telephone calls and semi-structured interviews were audio recorded with an encrypted voice recorder. Interviews were transcribed intelligent verbatim. A topic guide was created to support the interview process (Supplement 3).

### Sample size, randomisation and blinding

The study aimed to recruit 60 participants with an estimated dropout rate over 12 months of 15%, based on previously reported data [13]. Participants were randomised into intervention and control groups using an online block randomisation tool (envelope.com) [35]. The randomisation was stratified for cancer centre, cancer sites (colon or rectum), and no stratification was used for participants recruited via public domains. Participant and researcher were unblinded. Allocation concealment was overseen by an independent researcher, and independent researcher kept record of all participants data collected from the eligibility questionnaire prior to randomisation.

### Statistical analysis

Data were analysed using descriptive statistics—means, standard deviations and 95% confidence intervals. The last value carried forward method was used to impute the small number of missing data for body weight (for 6 participants at 3 months and for 3 participants at 6 months). For normally distributed data, a multiple regression model was used to explore the changes in potential main outcomes of the future trial. The outcomes used in the multiple linear regression models were WCRF/AICR score and the DQI-I, confounders included baseline score, age, gender, site, location and presence of a stoma. As this is a feasibility study with a relatively small sample size we did not pre-specify sensitivity analyses for exploratory multiple regression analysis as this is not warranted [36–38]. All data were analysed in STATA 15 (StataCorp, TX: StataCorp LLC) [39]. The data were considered significant with *p* ≤ 0.05. The transcribed interviews were uploaded to NVivo 12 software (QSR International Pty Ltd., Doncaster, VIC, Australia) and analysed using the five stages of framework analysis [40].

## Results

### Recruitment and randomisation

**Fig. 1:**
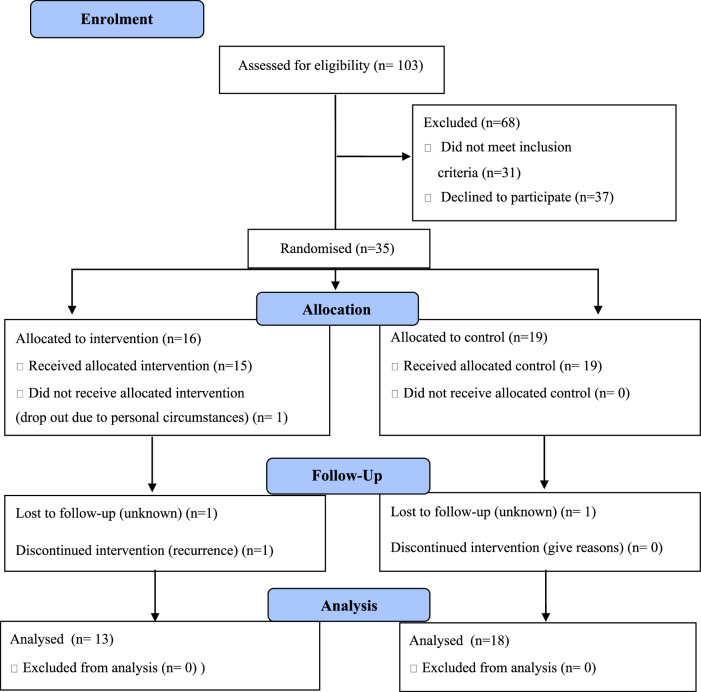
The Consolidated Standards of Reporting Trials (CONSORT) diagram.

Participants were recruited between 30 January 2021, and 15 December 2021. A total of 124 potential participants were approached, 96 via hospitals and 28 contacted the researcher directly. 103 patients confirmed interest and 72 were eligible for inclusion in the trial. Reasons for ineligibility included: underlying medical conditions [13], already following most of the WCRF/AICR guidelines [11], living outside of England [3], ongoing active treatment [3] and being more than five years post-surgery [1]. After further consideration, of the eligible group, 11 were not interested, 10 decided it was not the right time for them to take part, seven thought it was too much work, five thought they did not need support, and four did not want to state the reason. In total, 35 participants consented and were randomised into the intervention (*n* = 16) and control (*n* = 19) group. Recruitment rate over one year was 48.6%. See Fig. 1.

At baseline, the mean age of randomised participants was 65.9 years (SD 11.4, 95% CI 63.7 to 68.1) and ranged from 29 to 85 years. All participants were white British, and the majority lived in the North of England (*n* = 84, 80.0%). Nearly half of the participants had a degree or higher degree (45.7%) and were retired (51.4%). Most participants had had bowel cancer surgery (*n* = 24, 65.7%) and 20% have had a stoma (*n* = 28, 80%). Half of the participants were within the first-year post-surgery and treatment, and almost half of the participants underwent chemotherapy post-surgery (*n* = 15, 42.9%) (Table 1). Histological data were available for participants (*n* = 23, 65.7%) recruited via hospitals. Details are presented as Supplement 4.

**Table 1:** Participants’ characteristics at baseline.

		Intervention	Control
Characteristics	*N* = 35	*n* = 16	*n* = 19
	Mean	Mean	Mean (SD)
	(SD)	(SD)	
**Age (y)**	65.9 (11.4)	64.3 (13.3)	67.3 (9.7)
**Weight (kg)**	79.7 (15.6)	79.6 (11.5)	79.7 (18.7)
**BMI (kg/m**^**2**^**)**	27.6 (4.5)	27.1 (3.8)	27.9 (5.2)
**Waist circumference (cm)**	96.9 (12.3)	98.4 (11.3)	95.6 (13.2)
**Hip circumference (cm)**	104.9 (7.9)	105.5 (6.6)	104.5 (10.0)
	***n*** **(%)**	***n*** **(%)**	***n*** **(%)**
**Sex**			
Female	16 (45.7)	7 (43.8)	9 (47.4)
Male	19 (54.3)	9 (56.2)	10 (52.6)
**Ethnicity**			
White British	35 (100)	16 (100)	19 (100)
**Education**			
No formal education	2 (5.7)	0 (0)	2 (10.5)
Certificate	4 (11.4)	3 (18.8)	1 (5.3)
GCSE or A level	9 (25.7)	2 (12.5)	7 (36.4)
Diploma	4 (11.4)	2 (12.5)	2 (10.5)
Degree	10 (28.6)	7 (43.7)	3 (15.8)
Higher degree	6 (17.2)	2 (12.5)	4 (21.5)
**Monthly household income**			
Under £250	1 (2.8)	0 (0)	1 (5.3)
£501 to £1000	3 (8.6)	1 (6.3)	2 (10.5)
£1001 to £2000	7 (20.0)	4 (25.0)	3 (15.8)
Over 2000	19 (54.3)	8 (50.0)	11 (57.9)
No answer	5 (14.3)	3 (18.8)	2 (10.5)
**Marital status**			
Single	5 (14.3)	2 (12.5)	3 (15.8)
Married	23 (65.7)	12 (75.0)	11 (57.9)
Divorced	3 (8.6)	2 (12.5)	1 (5.3)
Widowed	4 (11.4)	0 (0)	4 (21.0)
**Work status**			
Working	18 (51.4)	9 (56.3)	9 (47.4)
Retired	19 (48.6)	7 (43.7)	10 (52.6)
**Location**			
North	28 (80.0)	12 (75.0)	16 (85.0)
Other	7 (20.0)	4 (25.0)	3 (15.0)
**Year of surgery**			
2016	1 (2.9)	1 (6.3)	0 (0)
2017	2 (5.7)	0 (0)	2 (10.5)
2018	6 (17.0)	2 (12.5)	4 (21.1)
2019	8 (22.9)	4 (25.0)	4 (21.1)
2020	9 (25.7)	5 (31.2)	4 (21.1)
2021	9 (25.7)	4 (25.0)	5 (26.2)
	***n*** **(%)**	***n*** **(%)**	***n*** **(%)**
**Surgery location**			
Bowel	23 (65.7)	12 (75.0)	13 (68.4)
Rectum	8 (22.9)	4 (25.0)	4 (21.1)
Both	4 (11.3)	0 (0)	2 (10.5)
**Stoma**			
No	28 (80)	13 (81.3)	15 (78.9)
Stoma	7 (20)	3 (18.7)	4 (21.1)
**Chemotherapy**			
Yes	20 (57.1)	11 (68.8)	9 (47.1)
No	15 (42.9)	5 (31.2)	10 (52.9)
**Smoking status**			
Never	18 (51.4)	9 (56.3)	9 (47.4)
Past	14 (40.0)	7 (43.8)	7 (36.8)
Present	3 (8.6)	0 (0)	3 (15.8)

### Feasibility outcomes

Retention rates post intervention were 97% (34/35) at three months and 89% (31/35) at six months follow up. One participant from the intervention group left the study within three months due to personal circumstances. A further three participants left the study during six months follow up. One participant had a recurrence of CRC, and two participants—one from the intervention group and one from control were lost to follow up. Data completion rates were high for food diaries (>90%) and questionnaires (>90%) but very low for pedometer data (40% of participants had lost their pedometer at six months). All remaining participants in the intervention group, completed all six telephone calls during the three months intervention and six telephone calls during the six months follow up period. The estimated time for completion of the study with three months intervention and six months follow up period was 39 weeks. The median time to complete the study was 41 weeks. Thus, the intervention was feasible for 13 out of 15 intervention participants (87%). During the study period one participant was diagnosed with skin cancer and two participants with lung cancer. One participant had a recurrence of residual colorectal cancer. One participant had a stroke. Survival rate at the end of the study was 100%.

### Acceptability of the intervention

The most frequently used booklets were Fruit and Vegetables (15/16), Wholegrain (13/16) and Physical Activity (13/16) booklets. Participants liked healthy tips and swaps, lists of fruit, vegetable, nuts/seeds and wholegrains based on fibre content, and list of exercises with pictures and description of how to perform them. Participants rated increasing fruit and vegetable intake and reducing fast food as the easiest changes at the start of the intervention (mean score 2.3 out of 5), while increasing wholegrain and reducing sugar intake were rated as the most difficult (mean 3.9 out of 5). Most participants (*n* = 14, 87.5%) chose to start with dietary booklets, while two (12.5%) preferred to make improvements in their physical activity first. On average, participants set five goals and achieved four of them. See Supplement 5. At six months follow up, participants had maintained the goals they had been practising at three months. On average, the confidence score was 8 and 8.4, respectively (1—the lowest, 10—the highest score).

Based on the interviews, the key themes were identified—the “acceptability of the intervention”, “traits for success”, and the “study wins”. The names of participants were replaced to maintain anonymity.

#### Acceptability of the intervention theme

Participants believed intervention had a positive impact on their health, commenting that it was “really good, and I think if it becomes a programme in the future, then I think, after surgery, people should be almost referred to do something like this.” [Rose]. Participants suggested changes in the delivery of the intervention such as reducing the amount of paperwork at the start of the intervention, and careful presentation of the study time frame. Also, three to six months post-intervention was suggested as the most appropriate time for most people to engage in lifestyle changes.

#### Traits for success theme

There was a unanimous message about needing further help with lifestyle post-surgery. Furthermore, participants were united about the need for conversation with a professional that served as an empathetic, reflective support, with the opportunity to ask questions: “I found it incredibly useful and, you know, it’s the first time, since before my surgery, that I’ve actually had someone I can talk to about, you know, what I eat and how I feel and, so, you know, in all sorts of ways, it’s been, it’s just been really a positive experience.” [Olivia] Also, the conversation with a professional was seen as the ability to practice a commitment to goals. The accountability of “given promise” or “shared plan” was seen by all participants as crucial in their success and effectiveness.

#### Study wins theme

Participants in the study reported various benefits, including feeling good about themselves and improving their skills in healthy shopping and food selection. Those with a stoma felt the program helped them better understand how their stoma functioned. Additionally, participants highlighted increased awareness about their habits, finding the study “eye-opening.” [Jacob] This awareness was fostered through activities such as reading booklets, wearing a pedometer, keeping food diaries, engaging in conversations, and receiving feedback. As a result, participants realised their previous misconceptions about healthy eating habits and recognised areas for improvement: “I thought to myself that I was eating healthy before I went on this, before I started this course. But I know now that I wasn’t, I wasn’t eating healthy…” [Harry].

The Shanyinde et al. [28] criteria for feasibility studies were used to evaluate the intervention [28]. The assessment is presented in Table 2. Based on the criteria reviewed on recruitment, proportion of eligible participants, randomisation, adherence to intervention, participant’s acceptability, outcome completeness, appropriateness, and retention rates, 10 out of 13 criteria were satisfactory and 3 out of 13 required attention. This was due to issues identified with the recruitment that might influence the ability to reach a sufficient sample size in a fully powered trial. Apart from recruitment the trial did not face any major challenges. Hence, based on the questions evaluated, the intervention was judged as being feasible to be performed as a definitive randomised trial.

**Table 2:**
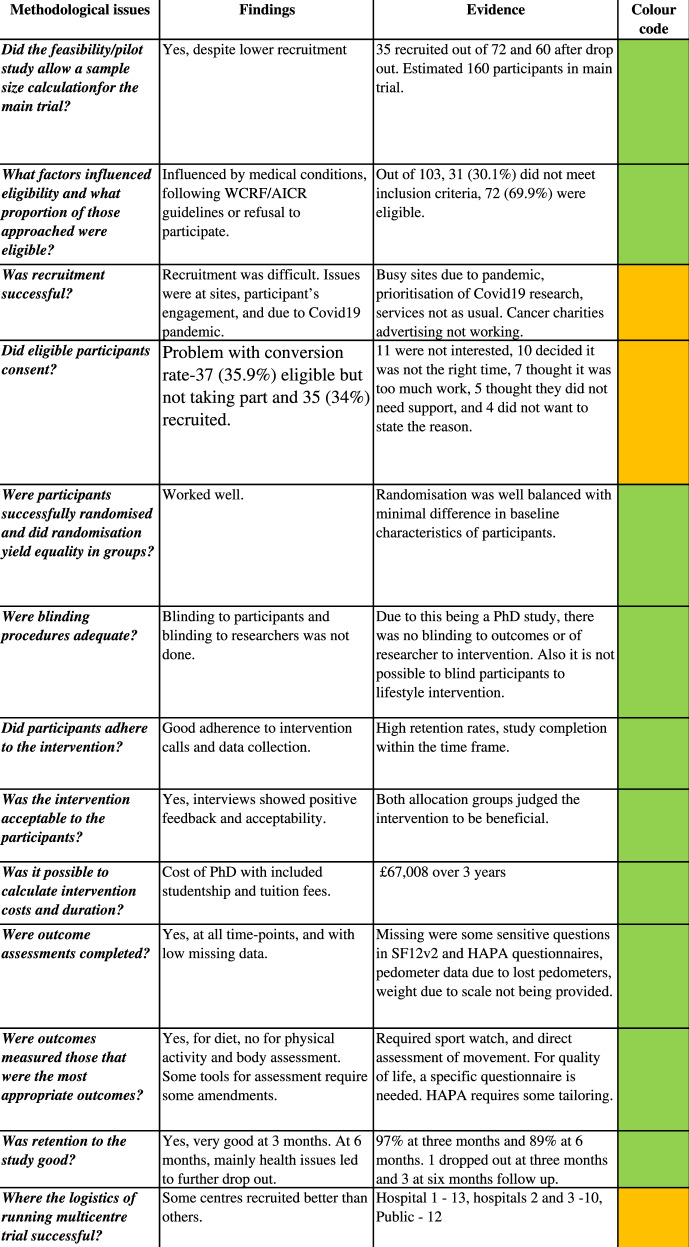
Progression criteria for feasibility trial.

Notes: The coloured panels represent a traffic light system to evaluate each question: green—achieved, orange—requires change, red—failed.

The mean WCRF/AICR adherence score was 4.1 (SD 1.1, 95%CI 3.7 to 4.5) at baseline, and was slightly, but not significantly, higher in the control group. At three months post intervention, the score improved only in the intervention group. At six months follow up, the score in the intervention group remained the same, and slightly improved in the control group. See Table 3. DQI-I score consistently increased in the intervention group but remained the same in the control group over time. The consumption of UPFs decreased below 30% of total energy intake per day in the intervention group, while in the control group intake increased to 45%. See Table 4. Data on anthropometry and physical activity are presented as Supplement 6 and 7. Multiple regression was performed to look at the potential primary outcomes in a fully powered trial. At three months and six months, the WCRF/AICR score and DQI-I score improved in the intervention group compared to control. See Table 5.

**Table 3:** Changes in World Cancer Research Fund/American Institute for Cancer Research score over time.

Time point	Baseline				3 months post intervention				6 months follow up			
Group	*N*/*n*	Mean	SD	95% CI	*N*/*n*	Mean	SD	95% CI	*N/n*	Mean	SD	95% CI
**Total**	35	4.1	1.1	3.7–4.5	34	4.7	1	4.3–5.1	31	4.7	1.1	4.3–5.1
**Intervention**	16	3.9	1.1	3.8–4.5	15	5.1	0.7	4.7–5.5	13	5.1	1.1	4.4–5.8
**Control**	19	4.3	1	3.8–4.8	19	4.3	1.1	3.8–4.8	18	4.5	1	4.0–5.0

The score ranges from 0 to 7 (higher score means better adherence).

**Table 4:** Dietary changes reported over time.

Time point		Baseline				3 months post intervention				6 months follow up			
Variable	Group	*N/n*	Mean	SD	95% CI	*N/n*	Mean	SD	95% CI	*N/n*	Mean	SD	95% CI
**F&V [g/d]**	Total	35	297.7	146.9	247.2, 348.1	34	367.3	223.3	289.3, 445.2	31	365.4	217.3	285.7, 445.1
	Interv	16	309.1	173.8	216.5, 401.8	15	478.8	253.6	338.4, 619.3	13	470.3	194.2	352.9, 587.7
	Control	19	288	124	228.3, 347.8	19	279.2	151.1	206.4, 352.0	18	289.7	205.5	187.5, 391.9
**F&V [portions/d]**	Total	35	3	1.5	2.5, 3.5	34	4	2.5	3.1, 4.8	31	4.1	2.6	3.2, 5.0
	Interv	16	3.2	1.7	2.2, 4.1	15	5.2	2.8	3.6, 6.7	13	5.3	2.3	3.9, 6.7
	Control	19	2.8	1.2	2.2, 3.4	19	3	1.7	2.2, 3.8	18	3.2	2.5	1.9, 4.5
**Wholegrains [g/d]**	Total	35	43	48.7	26.3, 59.7	34	76	63.6	53.8, 98.1	31	56.3	63	33.2, 79.4
	Interv	16	39.4	47.2	13.4, 64.0	15	108.1	65.2	71.9, 114.2	13	72.9	51.1	42.1, 103.8
	Control	19	46.1	51.1	21.5, 70.7	19	50.6	50.6	26.2, 75.0	18	44.3	69.3	9.9, 78.8
**Red meat [g/w]**	Total	35	230.9	339.6	114.3, 347.6	34	68	120.3	26.0, 110.0	31	134.1	187.3	65.4, 202.8
	Interv	16	268.8	329.2	19.1, 60.9	15	101.8	147.1	20.3, 183.2	13	137.8	152.8	45.5, 230.2
	Control	19	199	353.8	28.5, 369.5	19	41.3	89.4	−1.7, 84.4	18	131.4	213.1	25.4, 237.4
**Processed meat [g/w]**	Total	35	234.6	248.5	149.2, 320.0	34	128.2	198.5	58.0, 197.4	31	153.5	258	58.9, 248.1
	Interv	16	279.9	274.3	93.4, 444.2	15	44.1	120.6	−22.7, 110.8	13	63.7	78.7	16.1, 111.3
	Control	19	196.4	224.9	88.0, 304.9	19	194.6	224.3	86.5, 302.8	18	218.3	320.1	59.2, 377.5
**Energy from UPF [%]**	Total	35	40	16.8	34.2, 45.8	34	35.2	13	30.7, 39.7	31	38.2	14.2	32.9, 43.4
	Interv	16	40.5	15.6	32.2, 48.9	15	28.6	12.9	21.5, 35.8	13	28.5	7.8	23.8, 33.2
	Control	19	39.5	18.2	30.8, 48.3	19	40.4	10.7	35.3, 45.6	18	45.1	13.9	38.2, 52.0
**Sugary drinks [g/d]**	Total	35	195.1	364.4	69.9, 320.3	34	238.8	392.2	101.9, 375.7	31	213.8	318.5	96.9, 330.6
	Interv	16	191.8	317.3	22.7, 360.9	15	207.2	338.7	19.7, 394.8	13	189.2	243.4	42.1, 336.3
	Control	19	197.9	408.5	1.0, 394.7	19	263.7	437.4	52.9, 474.6	18	231.5	369.4	47.8, 415.2
**Added sugar [tsp/d]**	Total	35	9.7	7.3	7.2, 12.6	34	7.4	5.7	5.4, 9.4	31	9.4	7.3	6.7, 12.1
	Interv	16	11.9	7.5	7.9, 16.0	15	5.7	4.9	3.0, 8.4	13	6.4	4.4	3.7, 9.1
	Control	19	7.8	6.8	4.5, 11.1	19	8.8	6	5.9, 11.7	18	11.6	8.2	7.5, 15.7
**DQI-I**^**a**^	Total	35	54.5	7.8	51.8, 57.2	34	58.3	10.3	54.9, 62.1	31	61	11.1	56.9, 65.1
	Interv	16	55.8	8.3	51.4, 60.3	15	63.5	9	58.0, 69.0	13	66.8	10.1	60.7, 72.9
	Control	19	53.5	7.5	49.9, 57.1	19	54.6	8.9	50.3, 58.9	18	56.9	10.2	51.8, 61.9

*CI* Confidence intervals, *d* day, *DQI-I* Diet Quality Index International, *d* day, *g* gram, *F&V* fruit and vegetables, *Inter* Intervention, *UPF* ultra-processed food, *Tsp* teaspoon.

^a^The DQI-I score ranges from 0, the lowest to 100, the maximum score.

**Table 5:** Multiple regression for World Cancer Research Fund/American Institute for Cancer Research score and Diet Quality Index-International.

	3 months post-intervention					6 months follow-up				
WCRF/AICR score	Coefficient	Standard error	*P* value	95% CI		Coefficient	Standard error	*P* value	95% CI	
WCRF/AICR score baseline	0.6	0.1	0. 0001*	0.3	0.9	0.5	0.2	0.02*	0.1	0.9
Intervention group	1.1	0.3	0.0001*	0.5	1.6	0.9	0.4	0.03*	0.1	1.7
Age	0	0	0.5	0	0	0	0	0.9	0	0
Gender	0.3	0.3	0.4	−0.3	0.8	−0.3	0.4	0.4	−1.1	0.5
Site 2	1	0.4	0.02*	0.2	1.8	1	0.5	0.1	−0.1	2.1
Site 3	0.5	0.3	0.1	−0.2	1.2	0.7	0.5	0.2	−0.3	1.7
Location 2	−0.3	0.4	0.5	−1	0.5	−0.1	0.5	0.9	−1.1	1
Location 3	0.2	0.4	0.7	−0.8	1.1	−0.1	0.7	0.8	−1.5	1.2
Stoma	−0.2	0.4	0.7	−1	0.6	−0.1	0.6	0.8	−1.3	1
DQI-I score baseline	0.4	0.2	0.042*	0	0.7	0.3	0.2	0.2	−0.2	0.7
Intervention group	8.9	2.7	0.003*	3.3	14.5	8.2	3.4	0.026*	1.1	15.3
Age	0.4	0.1	0.011*	0.1	0.6	0.1	0.2	0.5	−0.3	0.6
Gender	1.2	2.8	0.7	−4.5	6.9	−5.6	3.6	0.1	−13.1	1.9
Site 2	−2.5	3.8	0.5	−10.4	5.5	4	4.8	0.4	−5.9	14
Site 3	−2.6	3.4	0.5	−9.5	4.4	−0.6	4.6	0.9	−10.3	9
Location 2	3.8	3.8	0.3	−4.1	11.7	5.9	4.8	0.2	−4.1	15.8
Location 3	0.9	4.3	0.8	−8	9.8	6.3	5.9	0.3	−6	18.6
Stoma	−11.5	4	0.009*	−19.9	−3.2	−11.4	5.2	0.041*	−22.2	−0.5

* indicates a significant differenc.

## Discussion

This was a feasibility randomised controlled trial testing the HEAL ABC study design and resources for people after CRC surgery and treatment.

Recruitment was the most challenging aspect of the intervention delivery. The study was completed with 35 participants and a recruitment rate of 48.6% over one year. Considering that the sample size of feasibility trials ranges from 10 to 300 participants per arm [41], this was a relatively small trial. However, based on previous recommendations [28, 42] the sample size met criteria for evaluation of feasibility. However, higher recruitment rates will be required to reach a sufficient sample size in a fully powered trial.

Compared to other UK studies, our recruitment rate was higher than seen for feasibility studies recruiting during COVID-19 pandemic [43], but lower than studies undertaken before the pandemic [44, 45]. Studies suggest that recruitment via hospitals and engagement of healthcare professionals can be problematic, and there can be low uptake from invitation letters [44–47]. In this study, invitation letters were less effective than anticipated. The hospitals engaged well with potential participants via phone to introduce the project and ask for permission to be contacted by researchers. However, the interest of those approached was low. We believe that direct contact with invested researcher helped increase the consent rate among those contacted. Also, the recruitment was supported by advertising via cancer charities. However, some participants explained during interviews that they avoid cancer-related charities and websites. Hence, recruitment should target primary and secondary care and the wider public domain.

Previously, only two RCTs with similar aims and design in patients after CRC have been completed [48, 49]. The limited number of lifestyle RCTs in the CRC population is likely related to problems with recruitment and participant engagement reported in this study and previously [43–46].

Also, current ethical committee requirements complicate the recruitment process, making it more difficult than necessary to maximise participant enrolment. Additionally, until recently, funders have not prioritised lifestyle interventions.

Therefore, simplifying the recruitment process by reducing paperwork and emphasising the benefits versus the effort required can likely help. Utilising smartphone technology for recruitment, consent, and data collection can potentially enhance participant uptake, reduce the burden and costs of data collection, and enable real-time data analysis from a large population over an extended period. For instance, in our study, older participants regularly used their smartphones but were less inclined to use computers.

Additionally, participation in a lifestyle intervention may be related to a participant’s belief regarding whether lifestyle impacts cancer development and recurrence [18, 50]. Based on our interviews people have limited knowledge about lifestyle and cancer recurrence.

Participants found the HEAL ABC intervention useful and highlighted that it should be part of the care pathway post-treatment. Interestingly, a good acceptability of the intervention was reported in all the feasibility studies conducted with people after CRC in the UK [43–46, 51]. Hence, it seems that despite challenging recruitment rates, participants who decide to participate find intervention beneficial and make improvements in their lifestyle.

Participants in the intervention group improved the mean WCRF/AICR adherence score by 1.2 points at three months and maintained the score at six months. Recently, a systematic review of 18 interventions that utilised WCRF/AICR adherence score showed that a 1-point increment in WCRF/AICR score is significant and leads to a reduction of 10% risk of overall cancer [52]. Hence, the improvement shown in this study may have a clinically meaningful effect. Around 57% of calories in the UK come from UPFs [53]. In this study, 40% of the energy consumed by participants came from UPFs at baseline, but it decreased to 28.5% in the intervention group at six months follow-up. Recent studies showed that every 10% increase in ultra-processed food in a diet leads to an increased incidence of cancer overall by 2% [54] and that substituting 10% of processed food and UPFs with an equal amount of minimally processed foods is associated with reduced overall cancer risk (HR 0·96, 95% CI 0.95–0.97) [55]. The HEAL ABC intervention did not directly focus on reducing UPFs in the diet. However, focusing on WCRF/AICR recommendations led to a 10% decrease in ultra-processed food intake, which based on these findings may lead to a reduction of overall cancer risk.

### Strengths and limitations

A theoretical foundation of the intervention, resources based on the theory and public and patients’ involvement in resources development are the main strengths of this research. The additional study strength is a telephone-based design with post or email communication and an extended follow-up period. Monitoring the trial, including fidelity assessment, intervention calls recording, and documentation of the feasibility trial, increased transparency and reproducibility. Additionally, the diet and physical activity were assessed using validated tools.

The main study limitations were a substantial population from Greater Manchester that prevents generalisability, prevalence of self-reported outcomes and limited blinding of participants and the personnel through data collection to analysis. Detection bias likely influenced reported outcomes in the control group because interviews revealed that repeated assessments were a motivator to make improvements, and the pedometers served as behavioural clue. Also, participants were not restricted to seeking help with diet and exercise outside the intervention, but this was monitored.

## Conclusion

The HEAL ABC intervention was feasible and delivered despite COVID-19 global pandemic. Recruitment was challenging and thus new strategies are needed to recruit people after CRC to RCTs. The HEAL ABC intervention was completed with a high retention rate and a low missing data. The intervention was acceptable to participants, positively rated and reported as beneficial. The intervention indicates that with supervision people after CRC can improve WCRF/AICR, DQI-I score, and reduce consumption of UPFs. However, a fully powered trial is needed to report on the effectiveness of the HEAL ABC intervention.

## Supplementary information

Supplementary materials
