# Supplementary material for: Healthy Eating and Active Lifestyle after Bowel Cancer (HEAL ABC)—feasibility randomised controlled trial
Source: Eur J Clin Nutr. 2024 Aug 27;78(12):1095–104. doi: 10.1038/s41430-024-01491-z (PMC11611738; doi:10.1038/s41430-024-01491-z)
Supplement: Supplementary file 1 — Supplementary materials [file 41430_2024_1491_MOESM1_ESM.pdf]

Supplementary materials

Supplement 1 Behaviour change techniques used

BCT no.*	Technique	HEAL ABC intervention	When used
1.2	Problem solving	Barriers are identified during the action planning process. Help to solve problems is provided during the supportive telephone calls.	Intervention
1.3	Goal settings (outcomes)	Outcome goals and incremental goals are sets at the start of each booklet participants follow.	Intervention
1.4	Action planning	An action plan is created at the start of each booklet participants follow.	Intervention
1.5	Review behaviour goal(s)	The participant's goal(s) are reviewed during supportive telephone calls every two weeks during the three-month intervention.	Intervention
1.7	Review outcome goal(s)	Every two weeks during the three-month intervention, and once a month during the six months' follow-up, the participant's goal(s) are reviewed during supportive telephone calls.	Intervention and follow up
2.2	Feedback on behaviour	Feedback on behaviour is provided every two weeks – during the supportive telephone calls during the three-month intervention and once a month during the six months' follow-up.	Intervention and follow up
2.7	Feedback on outcome(s) of behaviour	Feedback on outcome(s) of the behaviour is received at the end of the three-month intervention and six months' follow-up.	Intervention and follow up
3.2	Social support (practical)	Supportive calls are intended to guide participants on how to make changes in diet and physical activity, alongside the guide provided in the booklets.	Intervention and follow up
3.3	Social support (emotional)	Motivational interviewing technique informs the supportive telephone calls.	Intervention and follow up
4.1	Instruction on how to perform the behaviour	Tips, tricks and suggestions for change are detailed in each booklet, and additional support is provided during the telephone calls.	Intervention
5.1	Information about health consequences	Information about health consequences is detailed in each booklet participants follow.	Intervention
6.2	Social comparison	Participants assess their body, diet, and activity levels in the assessment booklet and compare them to healthy guidelines. Every booklet states healthy recommendations and explores participant's current habits.	Intervention
8.3	Habit formation	The follow-up booklet aims to support participants in habit formation and reinforce the repetition of changes achieved during the intervention. Telephone calls support the maintenance of change once a month during six months' follow up.	Follow up
8.7	Graded task	Participants follow one booklet at a time, setting incremental goals, gradually building self-efficacy and making changes during the intervention, and focusing on repetition of behaviour during the follow-up period.	Intervention and follow up
9.1	Credible source	The booklets are based on WCRF/AICR guidelines. The researcher is a certified nutritionist and fitness trainer, and has been trained in motivational interviewing techniques.	Intervention and follow up
9.2	Pros and cons	The pros and cons of behaviour are detailed in each booklet participants follow.	Intervention
12.6	Body changes	Body composition assessment, physical activity booklet, and exercise booklet aim to support participants in the body changes.	Intervention and follow up

Supplement 2 Control group leaflet

MANCHESTER 1824
The University of Manchester

HEAL ABC
Healthy Eating and Active Lifestyle After Bowel Cancer

Healthy Eating and Active Lifestyle After Bowel Cancer - HEAL ABC

Bowel cancer surgery or treatment can leave you feeling exhausted, but over time you get stronger and might want to shift your focus on your overall health. This can be a good time to take action towards improving your diet and activity level.

Even though, we cannot say with certainty that having healthy diet and active lifestyle will stop cancer from returning, improving your diet and activity level will help you prevent many diseases.

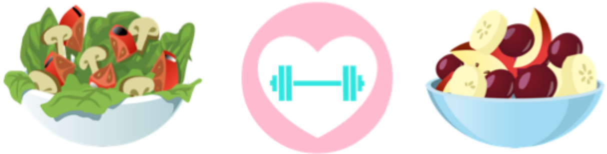

We invite you to look at the evidence based cancer prevention recommendations developed by ***World Cancer Research Fund and American Institute of Cancer Research*** recommended also for people after cancer:

Website link:
https://www.wcrf-uk.org/uk/preventing-cancer/cancer-prevention-recommendations

We encourage you to think about the prevention recommendations and take action towards making positive change in your diet and activity levels.

World Cancer Research Fund organisation has also wonderful website for healthy recipes:

Website link:
https://www.wcrf-uk.org/uk/recipes/recipes-home

You can find lot of helpful suggestions on **Bowel Cancer UK** website:

Website link:

https://www.bowelcanceruk.org.uk/about-bowel-cancer/living-with-and-beyond-bowel-cancer/

Bowel Cancer UK has a lovely booklet on diet and physical activity.

Website link:

https://bowelcancerorguk.s3.amazonaws.com/Publications/EatingWell_BowelCancerUK.pdf

Supplement 3 Topic guide

Intervention group - 3 months post intervention

BOOKLETS
Tell me your thoughts about the booklets?
Prompt: What was helpful about booklets? Would you change anything in the booklets?
What is the best time to change your diet and activity level after your surgery?
Prompt: Was it a right time for you to make changes in your lifestyle? Can you think of more appropriate time to start?

BEHAVIOUR
How did you find following different booklets?
How did you find setting up your goal?
How did you find planning your action?
How did you find tracking your action?
How did you find reviewing your goal/if they were reviewing the plan?
Prompt: Was it easy? Was it difficult? Did you have any others issues?
If person is overly positive about everything ask about

WHAT WAS THE MOST DIFFICULT?
Were 2 weeks enough for you to start making the change?
Have you experienced any struggles during the study? How did you cope with it?
What lifestyle changes did you do?

GUIDANCE
I really need a genuine feedback here. Please, be brutally honest with me.
What are your thoughts on the calls from the researcher?
Prompt: What did you like about the phone calls? What was unhelpful about the phone calls?
Would you expect more support? Do you have any other suggestion?

INTERVENTION
Tell me, what are your thoughts about the study?
How did you find the researcher visits to do data collection?
How did you find filling in the questionnaires?
How did you find 3 days dietary assessment?
How did you find using pedometer?

OUTSIDE OF HEAL ABC
Over the last 3 months have you attended any course or activity for healthy eating, weight loss or similar?
Over the last 3 months, have you attended any exercise programme?
Over the last 3 months, have you attended any mindfulness programme?
Do you practise mindfulness, mediation or praying?

OVERALL FEEDBACK
Overall, what do you think of the HEAL ABC programme?
Prompt: Was it helpful? Would you change anything? Have you used planning skills in another aspects of your life?

Would you recommend the HEAL ABC to others?
Prompt: Can you tell me why?
Is there anything else that you would like to comment on regarding your experience of the HEAL ABC programme?
Is there anything else you would like to tell me?

Control group - 3 months post intervention

WORLD CANCER RESEARCH FUND GUIDELINES
Have you looked at the guidelines?
Tell me your thoughts about the guideline?
Prompt: Were guidelines useful? Were guidelines helpful? Would you change anything in the guidelines?
What are your thoughts on the time when guidelines were introduced to you?
Prompt: Was it a right time to find out about it? Can you think of more appropriate time?

BEHAVIOUR
Have you used any suggestion from the guidelines? If yes, which one?
Prompt: What exactly did you do? How did you do it?
Did you make any lifestyle changes?

INTERVENTION
Tell me, what are your thoughts about the study?
Prompt: Was it positive experience? Was it negative experience?
How did you find the allocation to groups?
How did you find filling in the questionnaires?
How did you find 3 days dietary assessment?
How did you find tracking your steps?

OUTSIDE OF HEAL ABC
Over the last 3 months have you attended any course or activity for healthy eating, weight loss or similar?
Over the last 3 months, have you attended any exercise programme?
Over the last 3 months, have you attended any mindfulness programme?
Do you practise mindfulness, mediation or praying?

OVERALL FEEDBACK
Overall, what do you think of the study?
Prompt: Was it helpful? Would you change anything?
Is there anything else that you would like to comment on regarding your experience of being in the study?
Has anything else changed since you have been in the study that you want to tell me about?
Is there anything else you would like to tell me?

Intervention group - 6 months follow-up

BOOKLETS
Have you used booklet "My journey to better health?"
Tell me your thoughts about this booklet?
Prompt: did you make any notes? Was booklet useful? Was booklet helpful? Would you change anything in the booklet?

BEHAVIOUR

Continuation
Over the last six months,
…what diet changes have you sustained?
…what activity changes have you sustained?
Can you tell me about it? Can you tell me more?

What was your motivation to continue with the changes?
What helped you to continue with the changes?
What dietary changes you were not able to sustain?
What activity changes you were not able to sustain?

Goals
Have you set any new goal(s) during the last six months? Can you tell me about it?
How do you feel about setting a specific goal after being in the study? What does it mean to you?
How do you feel about planning the action after being in the study?

Barriers
Have you experienced any struggles to follow your plan? How did you cope with it?
What were your barriers to make a change / to continue?
Are there still any barriers that influence your diet?
Are there still any barriers that influence your activity level?

Evaluation
How successful do you think you were in making changes in your lifestyle on scale from 1 to 10?
What was benefit to participate in the study?
What were negatives in the study?

GUIDANCE
What are your thoughts on the calls from the researcher during the last six months?
Prompt: What did you like about the phone calls? Was there something you did not like about the phone calls? Would you expect more support? Do you have any other suggestion?

OUTSIDE OF HEAL ABC
Over the last 9 months have you attended any course or activity for healthy eating, weight loss or similar?
Over the last 9 months weeks have you attended any exercise programme?
Over the 9 months have you attended any mindfulness programme?
Do you practise mindfulness, regular mediation or praying?

OVERALL FEEDBACK
Overall, what do you think of the HEAL ABC programme?
Prompt: Was it helpful? Would you change anything? Have you used planning skills in another aspects of your life?
Would you recommend the HEAL ABC to others?
Is there anything else that you would like to comment on regarding your experience of the HEAL ABC programme? Can you tell me why?
Has anything else changed since doing the HEAL ABC that you want to tell me about?
Is there anything else you would like to tell me?

Control group - 6 months follow-up

WORLD CANCER RESEARCH FUND GUIDELINES
If they did not look at the guidelines at 3 months:
Have you looked at the guidelines?
Tell me your thoughts about the guideline?
Prompt: Were guidelines useful? Were guidelines helpful? Would you change anything in the guidelines?
If they looked at the guidelines at 3 months:
Have you looked at guidelines since we last spoke?

BEHAVIOUR
Have you used any suggestion from the guidelines? If yes, which one?
Prompt: What exactly did you do? How did you do it?
Did you make any lifestyle changes?
Did you continue with any lifestyle changes?
How successful do you think you were in making changes in your lifestyle?

INTERVENTION
Tell me, what is your overall experience to be in the study?

OUTSIDE OF HEAL ABC
Over the last 6 months, have you attended any course or activity for healthy eating, weight loss or similar?
Over the last 6 months, have you attended any exercise programme?
Over the last 6 months, have you attended any mindfulness programme?
Do you practise mindfulness, regular mediation or praying?

OVERALL FEEDBACK
Overall, what do you think of the study?
Prompt: helpful, anything changed, use planning skills in another aspects
Is there anything else that you would like to comment on regarding your experience of being in the study?
Has anything else changed since you have been in the study that you want to tell me about?
Is there anything else you would like to tell me?

Supplementary material 4 Histological data of study participants

Based on the medical records of participants recruited via hospitals (n=23). Histologically all participants had adenocarcinoma at different stages: T3 (n=7, 41.2%), T2 (n=4, 23.5%), T1 (n=2, 11.8%), T0 (n=2, 11.8%) or T4 (n=2, 11.8%). Most participants had had a right hemicolectomy (6, 25.0%), high anterior resection (5, 21.7%), left hemicolectomy (3, 12.5%), abdominal-perineal resection (3, 12.5%), end colostomy (3, 12.5%), low anterior resection (1, 4.2%), transverse colostomy (1, 4.2%), or sigmoid colectomy (1, 4.2%). Hypertension was the most frequent comorbidity (n=19, 54.3%), then hypercholesterolemia (n=6, 17%), type 2 diabetes (n=5, 14.3%), ischaemic heart diseases (n=5, 14.3%), asthma (n=4, 11.4%), hiatus hernia (n=3, 8.6%), inguinal or umbilical hernia (n=3, 8.6%), obesity (n=2, 5.7%) and arthritis (n=2, 5.7%).

Supplement 5 Goal Settings

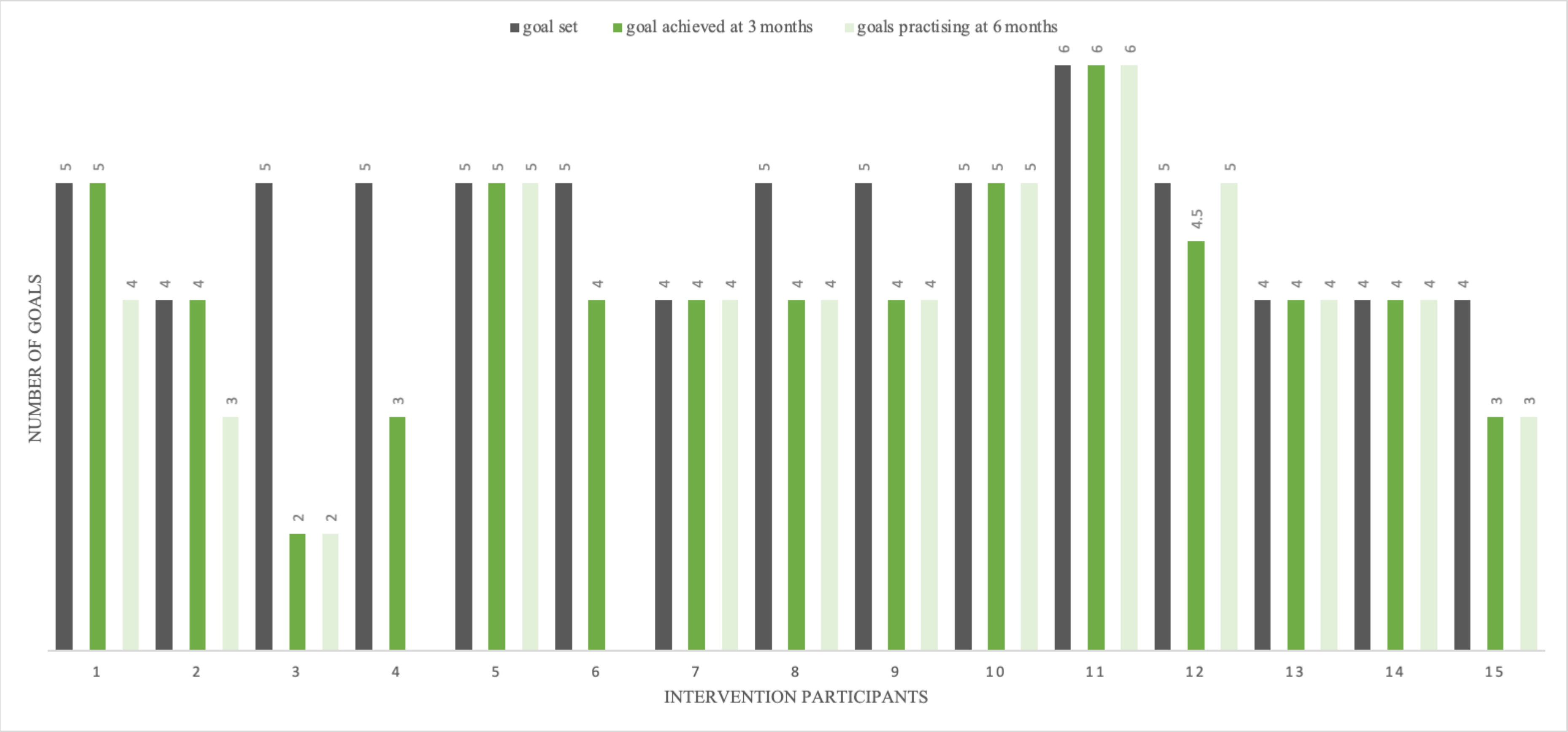

Supplement 6 Anthropometry changes over time

Time point		Baseline				3 months post intervention				6 months follow up			
**Variable**	**Group**	**N/n**	**Mean**	**SD**	**95% CI**	**N/n**	**Mean**	**SD**	**95% CI**	**N/n**	**Mean**	**SD**	**95% CI**
**Weight [kg]**	Total	35	79.7	15.6	74.3, 85.0	29	78.6	16.0	72.5, 84.7	28	76.3	12.5	71.5, 81.2
	Interv	16	79.6	11.5	73.6, 85.8	13	78.3	11.1	71.6, 85.0	13	77.6	11.5	59.0, 95.0
	Control	19	79.7	18.7	70.7, 88.7	16	78.8	19.5	68.44, 89.2	15	75.2	13.6	54.0, 104.0
**BMI [kg/m^2]**	Total	35	27.7	4.4	26.2, 29.2	29	27.1	4.7	25.3, 28.9	28	26.9	3.2	25.7, 28.1
	Interv	16	27.4	3.5	25.5, 29.1	13	26.5	3.6	24.3, 28.7	13	26.9	3.1	22.7, 32.4
	Control	19	28.0	5.1	25.5, 30.5	16	27.6	5.5	24.7, 30.6	15	26.9	3.4	20.7, 32.9
**Waist [cm]**	Total	35	96.9	12.3	92.4, 101.1	34	94.3	12.5	89.9, 98.7	31	93.4	11.5	89.1, 97.6
	Interv	16	98.4	11.3	92.4, 104.4	16	94.7	10.3	89.0, 100.4	13	95.2	12.2	76.2, 111.8
	Control	19	95.6	13.2	89.2, 101.9	19	93.9	14.3	87.0, 100.8	18	92.0	11.2	73.7, 116.8
**Hip [cm]**	Total	35	104.9	7.9	102.4, 107.7	34	104.2	8.4	101.3, 107.2	31	103.2	6.9	100.7, 105.8
	Interv	16	105.5	6.6	102.3, 109.1	15	103.6	7.3	99.6, 107.7	13	103.7	8.0	91.4, 120.1
	Control	19	104.5	10.0	100.2, 108.8	19	104.7	9.3	100.2, 109.2	18	102.9	6.3	91.4, 116.8

Supplement 7 Physical activity changes over time assessed with GPAQ and pedometer

Time point		Baseline				3 months post intervention				6 months follow up			
Variable	Group	N/n	Mean	SD	95% CI	N/n	Mean	SD	95% CI	N/n	Mean	SD	95% CI
**Activity [min/w]**	Total	35	755.8	616.2	544.2, 967.5	34	618.1	436.8	465.7, 770.5	30	954.0	454.6	784.3, 1123.8
	Interv	16	863.1	777.2	449.0, 1277.2	15	628.7	454.5	377.0, 880.4	13	1028.7	489.8	732.7, 1324.6
	Control	19	665.5	441.5	452.7, 878.3	19	609.7	434.6	400.3, 819.2	17	896.9	432.1	674.8, 1119.1
**Walking [min/w]**	Total	35	392.0	349.1	272.0, 511.8	34	306.2	210.7	232.7, 379.7	30	522.1	334.1	397.4, 646.9
	Interv	16	482.8	430.9	253.2, 712.4	15	276.7	171.5	181.7, 371.6	13	548.2	386.8	314.5, 782.0
	Control	19	315.0	249.0	195.4, 435.4	19	329.5	239.3	214.1, 444.8	17	502.2	298.5	348.7, 655.6
**Moderate [min/w]**	Total	35	350.4	453.7	194.6, 506.3	34	307.4	344.8	187.0, 427.7	30	408.3	314.8	290.8, 525.9
	Interv	16	352.2	478.9	97.0, 607.4	15	349.4	399.4	128.2, 570.6	13	426.6	228.8	288.4, 564.9
	Control	19	348.9	444.7	134.6, 563.3	19	274.2	302.1	128.6, 419.8	17	394.3	374.0	202.0, 586.6
**Vigorous [min/w]**	Total	35	15.1	33.9	3.4, 26.7	34	36.8	106.3	-0.2, 73.9	30	48.9	114.9	6.0, 91.8
	Interv	16	15.0	41.0	3.4, 26.7	15	62.5	148.7	-19.8, 144.8	13	46.9	79.9	-1.4, 95.2
	Control	19	15.1	27.8	1.7, 28.5	19	16.6	50.2	-7.6, 40.8	17	50.4	138.4	-20.7, 121.6
**Sedentary [min/d]**	Total	35	419.1	188.7	354.3, 484.0	34	355.3	178.8	292.9, 417.7	30	364.8	131.3	315.8, 413.9
	Interv	16	459.4	199.8	352.9, 565.8	15	339.3	168.2	246.2, 432.5	13	364.2	132.7	284.0, 444.4
	Control	19	385.3	177.0	300.0, 470.6	19	367.9	190.2	276.2, 459.6	17	365.3	134.3	296.3, 434.3
**Steps [day]***	Total	34	5355	4438.6	3806, 6904	30	5727	4629.1	3998, 7455	21	6330	4334.3	4357, 8303
	Interv	15	6136	4566.8	4209, 8666	13	5835	260.4	3093, 8578	7	7185	3625.3	3832, 10538
	Control	19	4738	4357.8	263.8, 6838	17	5644	4834.9	3158, 8129	14	5902	4716.4	4716, 8626
**Time [min/d]***	Total	33	49.1	41.2	34.5, 63.7	29	51.8	41.7	36.0, 67.7	21	100.3	187.1	15.1, 185.4
	Interv	16	54.7	44.1	29.3, 80.2	12	51.3	34.8	29.2, 73.5	7	73.1	35.2	40.5, 105.6
	Control	19	44.9	39.7	25.8, 64.0	17	52.2	47.1	28.9, 76.4	14	113.8	229.5	-18.7, 246.4

Note**:** d - day; Interv- intervention, min – minutes, w – week, * data reported by pedometer
